# Neurointensive care results and risk factors for unfavorable outcome in aneurysmatic SAH: a comparison of two age groups

**DOI:** 10.1007/s00701-021-04731-4

**Published:** 2021-01-29

**Authors:** Vilja Välimäki, Teemu Luostarinen, Jarno Satopää, Rahul Raj, Jyri J. Virta

**Affiliations:** 1 Department of Neurosurgery, Helsinki University Hospital and University of Helsinki, Helsinki, Finland; 2 Division of Anesthesiology, Department of Anesthesiology, Intensive Care and Pain Medicine, University of Helsinki and Helsinki University Hospital, Helsinki, Finland

**Keywords:** Subarachnoid hemorrhage, Prognosis, Elderly, Risk factor

## Abstract

**Background:**

The mean age of actively treated subarachnoid hemorrhage (SAH) patients is increasing. We aimed to compare outcomes and prognostic factors between older and younger SAH patients.

**Methods:**

A retrospective single-center analysis of aneurysmal SAH patients admitted to a neuro-ICU during 2014–2019. We defined older patients as ≥70 years and younger patients as <70 years. For every older patient, we identified three younger patients with the same World Federation of Neurological Surgeons (WFNS) grade. We only included patients receiving active aneurysm treatment. Favorable functional outcome, defined as a Glasgow Outcome Scale (GOS) of 4–5 at 12 months, was our primary outcome. We used logistic regression to compare prognostic factors between the groups.

**Results:**

Ninety-five (85%) of 112 older patients and 317 (94%) of 336 younger patients received aneurysm treatment. Of the younger patients, 91% with a good-grade SAH (WFNS I-III) had a favorable outcome compared to 52% in the older good-grade SAH group. In poor-grade patients (WFNS IV-V), favorable outcome was seen in 51% of younger patients, compared to 24% of older patients. Acute hydrocephalus and intracerebral hemorrhage were associated with unfavorable outcome in the younger (OR 4.7, 95% CI 2.6–8.4, and OR 3.7, 95% CI 2.1–6.4), but not in the older patients (OR 1.8, 95% CI 0.8–4.2, and OR 1.3, 95% CI 0.5–3.1, respectively).

**Conclusions:**

In actively treated SAH patients, age was a major determinant of outcome. Factors reflecting increases in intracranial pressure associated with outcome only among younger patients.

**Supplementary Information:**

The online version contains supplementary material available at 10.1007/s00701-021-04731-4.

## Introduction

The increasing number of elderly people with good functional capacity has led to an increase in the number of elderly patients treated at intensive care units (ICU) [1, 11]. As a vascular disorder with lifelong modifiable risk factors [2, 18], subarachnoid hemorrhage (SAH) has been shown to reach its peak incidence at 70–75 years of age [17]. Therefore, the number of elderly patients with SAH treated at neuro-ICUs is increasing.

Few studies have compared outcomes between younger and older SAH patients [4, 26] or compared prognostic factors between age groups [6]. Additionally, two landmark studies on prognostic factors of hospitalized patients with aneurysmatic SAH (aSAH) have included relatively young patients with mean ages of 58 and 53 years, respectively [13, 24]. Additionally, in these studies, none or a minority of aneurysms received endovascular treatment, whereas today, endovascular treatment is selected in up to two thirds of aneurysms [14].

Our aim was to compare the outcomes of younger and older aSAH patients admitted to a neuro-ICU in an institution where most ruptured aneurysms receive endovascular treatment and to assess whether the prognostic factors for functional outcome and mortality at 12 months differ between the age groups. We chose 70 years of age as the cut-off point. We hypothesized that older age is an independent risk factor for poor outcome and mortality even when comparing patients with similar SAH severity.

## Methods

### Study setting and population

We conducted a single-center retrospective study of consecutive aSAH patients admitted to the neuro-ICU of the Helsinki University Hospital. The Helsinki University Hospital covers a population of approximately 2 million people. Finland’s health care system offers equal and low-cost public health care for all citizens regardless of e.g. income or insurance status. The neuro-ICU at Helsinki University Hospital is the only ICU providing neurosurgical and neurointensive care for citizens living in the hospital’s catchment area. Thus, all actively treated aSAH patients are transferred to the designated neuro-ICU.

We screened all patients admitted to the neuro-ICU and identified those with an International Statistical Classification of Diseases and Related Health Problems (ICD) 10 code of I60.0-I60.9 (including non-traumatic SAH from specific arteries and unspecified non-traumatic SAH) between January 2014 and May 2019. We then evaluated all patients and included only those with a verified aSAH and excluded those with other disorders.

**Fig. 1:**
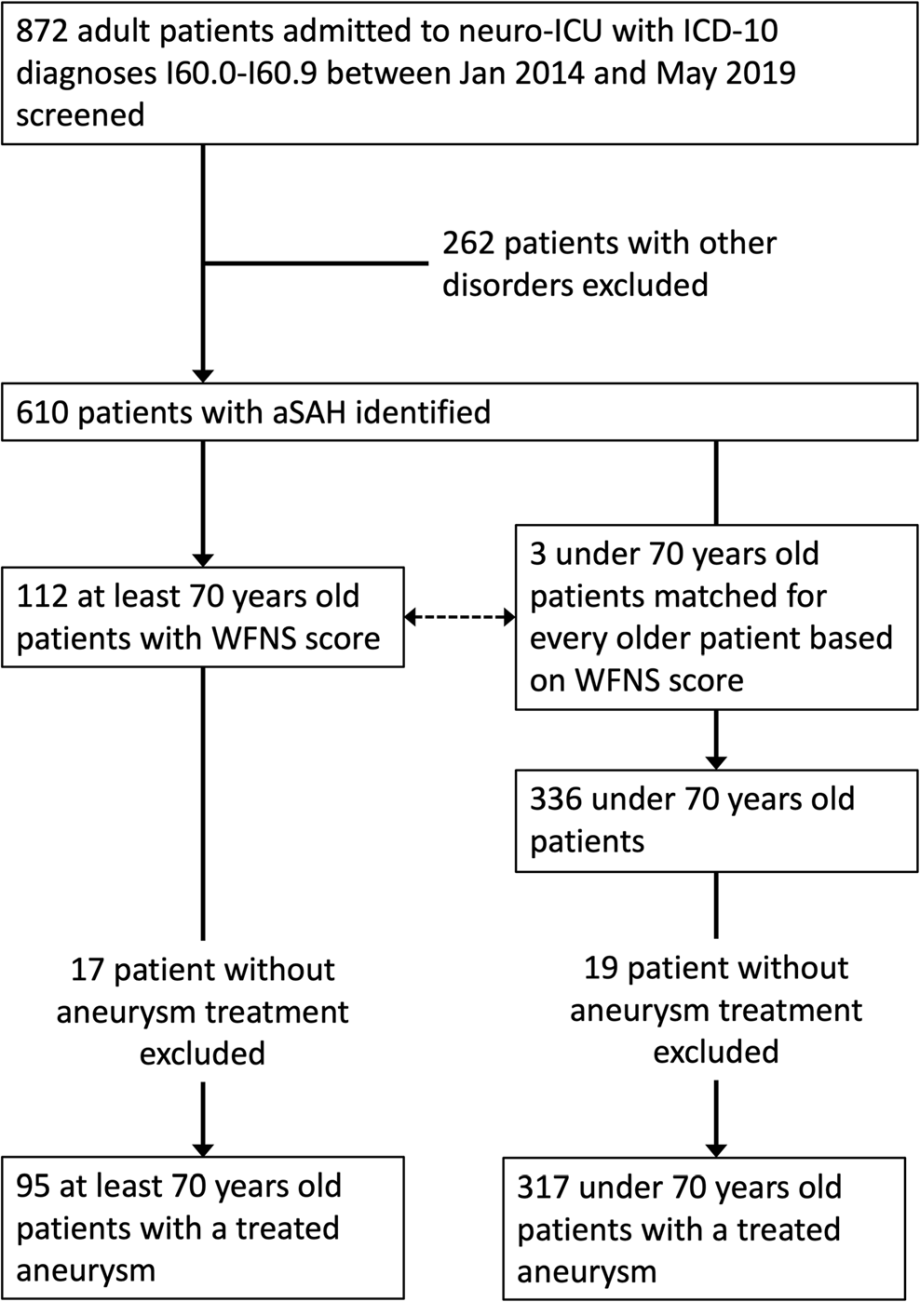
Study flow-chart. ICD-10: International Classification of Diseases. WFNS: World Federation of Neurological Surgeons scale

We then identified all ≥70-year-old patients with an available World Federation of Neurological Surgeons (WFNS) grade [7], excluding those who were initially admitted to the neuro-ICU only as potential organ donors. For every ≥70-year-old patient, we randomly selected three <70-year-old patients with a matching WFNS grade (i.e., 1:3 matching). This resulted in two groups of patients with an equal distribution of SAH severity, according to the WFNS grade. We refer to the ≥70-year-old patients as the older group and to the <70 years old as the younger group. A study flow chart is shown in Fig. 1.

### Data collection

After selecting patients to both groups, we scrutinized electronic health records and imaging data to extract the following variables: age at admission, Charlson Comorbidity Index (CCI) [5], use of antithrombotic medication (antiplatelets or anticoagulants), time from ictus to admission (< 12 h, 12–24 h or > 24 h), SAH severity using WFNS grading scale, location of the ruptured aneurysm (anterior or posterior circulation), aneurysm size, aneurysm treatment modality (surgical or endovascular), discharge unit from the neuro-ICU (step-down unit or ward), and presence of acute hydrocephalus requiring treatment with an external ventricular drain (EVD). For those patients who were intubated before admission at our hospital, we used the last recorded Glasgow Coma Scale (GCS) [27] score to estimate the WFNS grade.

From the admission computed tomography (CT) scan, we evaluated the presence of a thick and diffuse bleeding pattern (clot thickness ≥4 mm in ≥3 cisterns), any intraventricular hemorrhage (IVH) regardless of volume or number of ventricles affected, and intracerebral hemorrhage (ICH).

Additionally, we collected data on possible development of delayed cerebral ischemia (DCI) or shunt-dependent chronic post-SAH hydrocephalus. We diagnosed DCI based upon clinical symptoms and/or radiological findings. If a patient developed new neurological deficits not attributable to a previous focal lesion with radiological vasospasm on CT angiography (CTA), we initiated DCI treatment (intravenous nimodipine infusion and augmented hypertension) if feasible. If reliable neurological assessment was not possible due to intubation and severe radiological vasospasm was observed, we initiated treatment. If reliable neurological assessment was possible, the presence of radiological vasospasm without clinical findings did often not justify treatment. If a patient developed new CT hypodensities outside the direct vicinity of a previous focal lesion, we considered it a sign of DCI and initiated treatment.

We classified possible limitation-of-care orders during the stay in the neuro-ICU into three groups: do-not-resuscitate (DNR), weaning of life-supportive care, and treatment as a possible organ donor. Only the gravest limitation was recorded. Finland has an opt-out organ donation policy, with all patients having the possibility to act as organ donors if they are not known to have previously opposed organ donation.

### Outcomes

Our primary outcome was functional outcome at 12 months after aSAH determined retrospectively from expansive communal electronic health records. We defined favorable outcome as Glasgow Outcome Scale (GOS) [15] 4–5 and unfavorable outcome as GOS 1–3. We also recorded whether the patients were able to live at home (with or without external help) or had to be institutionalized (e.g., in a habilitation facility or hospital) at 12 months. Our secondary outcome was all-cause mortality at 12 months after aSAH.

### Statistical analyses

As age, aneurysm size, and duration of neuro-ICU treatment were not normally distributed (according to Shapiro-Wilk test and visual inspection of histograms), we report median and interquartile range (IQR) values for the continuous variables. For categorical variables, we report frequencies. We divided CCI into three categories: 0, 1, and ≥ 2 points. For risk factor analyses, we dichotomized SAH severity into good and poor grades (WFNS I-III and WFNS IV-V, respectively). In the outcome and risk factor analyses, we included only patients who had their aneurysm secured.

Due to the matched nature of our data, we used a generalized mixed model when comparing the age groups, using age group as a fixed effect and matching group (including one older patient and three WFNS-matched younger patients) as a random effect.

To assess risk factors for unfavorable outcome and all-cause mortality, we performed univariate logistic regression analyses separately for both age groups yielding odds ratios (OR) with 95% confidence intervals (CI). Reference group for each factor is described in the results. Additionally, to further assess possible interactions between age and the effect of potential risk factors, we performed logistic regression analyses for all patients including a potential risk factor, age group (younger vs. older group), and their interaction term (i.e., risk factor × age group).

To test whether age was an independent risk factor for unfavorable functional outcome and mortality at 12 months, we performed a multivariate logistic regression analysis on all patients. Based on the univariate analyses and clinical correlation, the multivariate model included CCI, SAH severity, presence of acute hydrocephalus, presence of IVH, presence of ICH, presence of a thick and diffuse SAH, and aneurysm location [13, 24]. We then added age group to this original model. We report Nagelkerke *R*^2^ for both models. If age group was significantly associated with outcome in this new model, and if the new model explained more of the variance in the outcome (i.e., if the difference between the Log-likehoods between the models was significant), we considered age group to be independently associated with our outcome.

As we expected there to be few missing values, we excluded those with missing values from the comparison analyses. We considered *p* values <0.05 as statistically significant. As statistical power to detect interactions is lower than for main effects [3, 8], we did not use the significance threshold of *p* < 0.05 for the interaction term. An age group × risk factor effect was considered possible, if the ORs differed between the age groups and the *p* value of the interaction term was <0.10.

### Ethics approval

The local institutional research committee approved the study and waived the need for patient consent (HUS/466/2019 §106). The study was conducted according to the Strengthening the Reporting of Observational studies in Epidemiology (STROBE) Statement.

## Results

### Population characteristics

Overall, we identified 610 aSAH patients admitted to the neuro-ICU between January 2014 and May 2019. The median age of all patients was 57.8 years (IQR 49.6–67.4). The WFNS distribution of all admitted aSAH patients did not differ between the age groups (Mann-Whitney *U* test, *p* = 0.48), and the proportion of poor-grade aSAH patients did not differ between younger and older patients (40% vs. 40.5%, respectively, *p* = 0.96). The mean age of patients did not change during the study period (*p* = 0.87).

After excluding 4 patients who were initially admitted as potential organ donors, there were 112 patients over the age of 70 years with available WFNS data. Hence, 336 patients under the age of 70 years were selected for the analyses matching for WFNS grade.

Seventeen (15%) of 112 patients in the older group did not have their aneurysm secured because the treating clinicians regarded their prognosis too poor. In contrast, only 19 (6%) of 336 patients in the younger group did not have their aneurysm secured. The difference between the groups was significant (*p* = 0.002). Overall, this left 412 patients for our analyses (317 in the younger group and 95 in the older group). The study flow chart is shown in Fig. 1.

### Group characteristics

Even after excluding patients whose aneurysm was not secured, the WFNS distribution was similar between the age groups. The median age was 55 years (IQR 48–63) in the younger group, and 75 years (IQR 72–80) in the older group (difference between groups significant, *p* < 0.001). The older patients were more often women, had a higher CCI score, and were more often on antiplatelet or anticoagulant medication. Additionally, they were more likely to have a thick and diffuse bleeding pattern. There was no difference in the presence of acute hydrocephalus, ICH, or IVH between the groups.

The age groups did not differ in aneurysm location, size (median maximal diameter 6.0 mm [IQR 5.0–9.0] in the younger group, and 6.0 mm [IQR 4.0–9.0] in the older group, *p* = 0.99), or treatment modality.

When considering only the 404 patients alive after 1 week, DCI was more often diagnosed and treated in the younger group. Among the 393 patients alive 2 weeks after admission, the older patients were more likely to develop chronic hydrocephalus than younger patients.

Limitation-of-care orders were more common among the older. The duration of neuro-ICU treatment (including possible readmissions) was longer in the younger group (median 12 days, IQR 7–16) than in the older group (median 9 days, IQR 4–15, *p* < 0.001). The older patients were more often discharged to a step-down unit instead of a regular ward.

Detailed group characteristics and comparisons are shown in Table 1.

**Table 1:** Demographic, clinical, and radiologic characteristics of the 412 patients included in the study

	Younger group (*N* = 317)		Older group (*N* = 95)		*p*
	*n*	%	*n*	%	
Sex					< 0.001
Women	187	59.0	81	85.3	
Men	130	41.0	14	14.7	
Charlson comorbidity index					<0.001
0	253	79.8	55	58.5	
1	47	14.8	29	30.9	
At least 2	17	5.4	10	10.6	
Antithrombotic medication					<0.001
None	298	94.0	68	71.6	
Antiplatelet	7	2.2	17	17.9	
Anticoagulant	12	3.8	10	10.5	
Delay from ictus to admission					0.15
Under 12 h	223	70.6	59	62.1	
12–24 h	22	7.0	12	12.6	
Over 24 h	71	22.5	24	25.3	
WFNS scale					0.27
I	120	37.9	37	38.9	
II	70	22.1	18	18.9	
III	17	5.4	10	10.5	
IV	44	13.9	11	11.6	
V	66	20.8	19	20.0	
SAH characteristics					
Thick and diffuse clot	158	49.8	26	27.4	<0.001
Intraventricular hemorrhage	174	54.9	59	62.1	0.20
Intracerebral hemorrhage	104	32.8	31	32.6	0.98
Aneurysm location					0.17
Anterior circulation	275	86.8	77	81.1	
Posterior circulation	42	13.2	18	18.9	
Treatment modality					0.06
Endovascular	161	50.8	59	62.1	
Surgical	156	49.2	36	37.9	
Subarachnoid hemorrhage complications					
Acute hydrocephalus	145	45.7	53	55.8	0.08
Delayed cerebral ischaemia^a^	156	50.0	24	26.1	<0.001
Chronic hydrocephalus^b^	72	23.5	31	36.0	0.02
Limitation-of-care orders					<0.001
None	299	94.3	73	76.8	
Do-not-resuscitate	4	1.3	15	15.8	
Weaning of life-supportive care	8	2.5	7	7.4	
Treated as a potential organ donor	6	1.9	0	0.0	
Discharge from neurointensive care unit					<0.001
Ward	207	65.5	39	41.1	
Step-down unit	101	32.0	53	55.8	
Died at the neuro-ICU	8	2.5	3	3.2	
Outcome at 12 months^c^					<0.001
Favorable outcome	241	77.0	40	43.0	
Unfavorable outcome	72	23.0	53	57.0	

^a^Only those alive after 1 week, *n* = 404

^b^Only those alive after 2 weeks, *n* = 393

^c^Only those with 12-month outcome data available, *n* = 406

*WFNS*, World Federation of Neurosurgical Societies grading scale

### 12-month mortality and functional outcome

Twenty-two (7%) of the 317 younger patients died during the first 12 months, whereas 26 (27%) of the 95 older patients died (*p* < 0.001).

Twelve-month functional outcome was available for 406 patients. For those alive at 12 months, the follow-up time was shorter in the younger group (median 329 days, IQR 231–389 days, and median 357 days, IQR 295–472 days in the older group, *p* < 0.001).

**Fig. 2:**
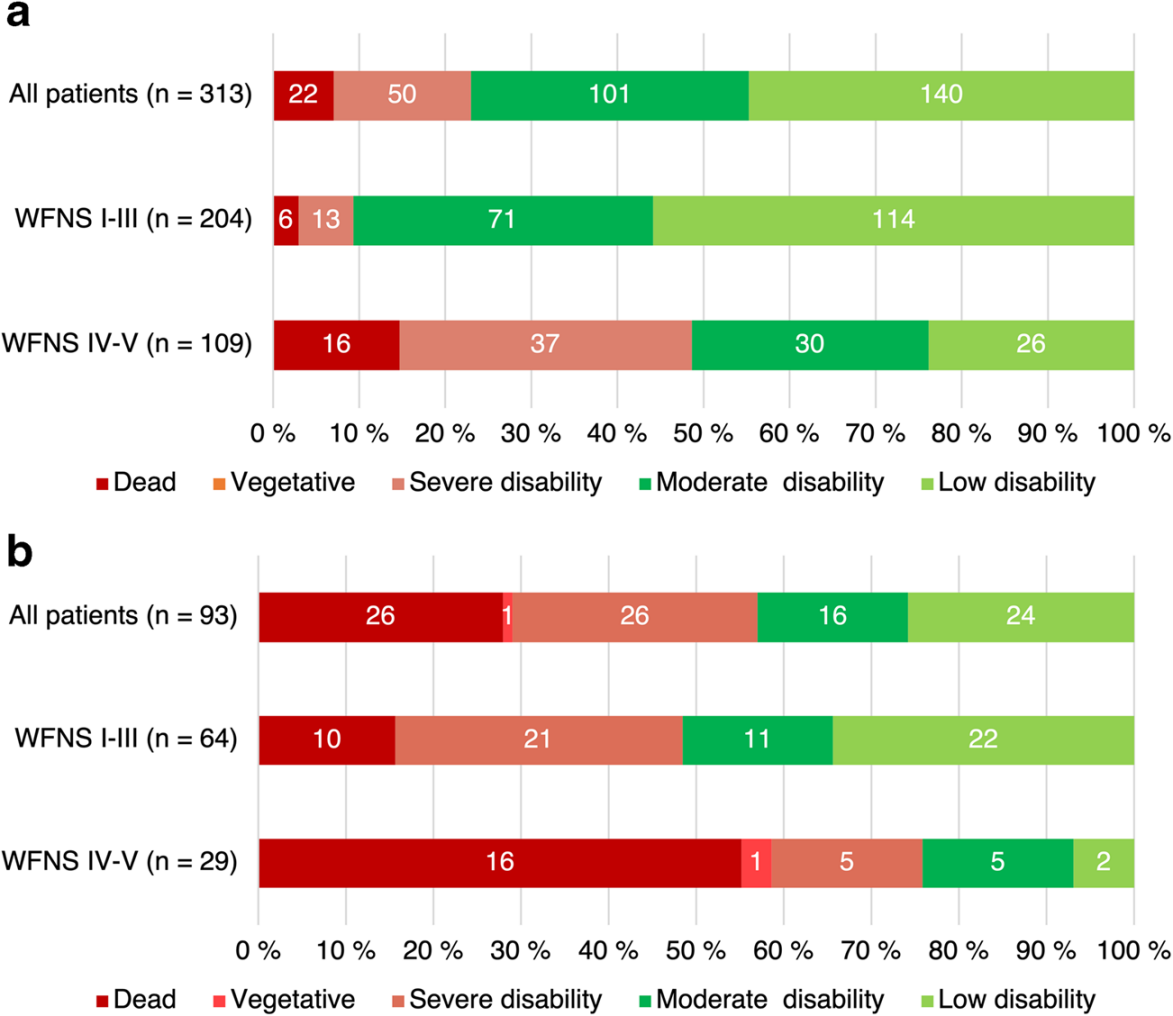
Distribution of functional outcomes at 12 months according to Glasgow Outcome Scale. Distributions are shown for all patients and separately for good-grade (World Federation of Neurological Surgeons scale [WFNS] I–III) and poor-grade (WFNS IV–V) patients. The numbers in the bars indicate the number of patients in each group. Favorable outcome has a green background and unfavorable a red background. **a** Younger patients (under 70 years of age). **b** Older patients (at least 70 years of age)

Two hundred forty-one (77%) of the 313 younger patients had a favorable outcome compared to 40 of 93 patients (43%) in the older group (*p* < 0.001). Of those alive at 12 months, 93% of the younger patients and 82% of the older patients were living at home (*p* = 0.02). Detailed functional outcome information is shown in Fig. 2.

### Risk factors for unfavorable outcome at 12 months

Sex or CCI was not associated with unfavorable outcome in either age group. Poor-grade SAH was associated with unfavorable outcome in both groups, but the association was stronger in the younger patients. Acute hydrocephalus was associated with unfavorable outcome only in the younger group. ICH was associated with unfavorable outcome in the younger group, but not in the older group and the interaction with age group seemed significant. IVH was associated with unfavorable outcome only in the younger group, but the interaction with age group did not seem significant. A thick and diffuse bleeding pattern was not associated with functional outcome in either group.

Surgical aneurysm treatment was associated with unfavorable outcome compared to endovascular treatment in the older group but not in the younger group. The interaction with age group seemed significant. A posterior circulation aneurysm was associated with unfavorable outcome in the younger group compared to an anterior circulation aneurysm, but not in the older group. Still, the interaction with age group was not significant. Results of the risk factor analyses are shown in Table 2.

**Table 2:** Results of the univariate logistic and interaction analysis for outcome at 12 months after subarachnoid hemorrhage. Proportion of patients with favorable and unfavorable outcomes as well as odds ratios (OR) with 95% confidence intervals (CI) for unfavorable outcome are shown

	Younger group (*n* = 313)				Older group (*n* = 93)				Age group interaction (*p*)
	Favorable (%)	Unfavorable (%)	OR	95% CI	Favorable (%)	Unfavorable (%)	OR	95% CI	
Sex									
Women	78.3%	21.7%	1.00		43.0%	57.0%	1.00		0.80
Men	75.2%	24.8%	1.19	0.70–2.02	42.9%	57.1%	1.01	0.32–3.18	
CCI									
0	79.3%	20.7%	1.00		46.3%	53.7%	1.00		0.69
1	66.7%	33.3%	1.91	0.96–3.82	41.4%	58.6%	1.22	0.49–3.04	
At least 2	70.6%	29.4%	1.60	0.54–4.73	30.0%	70.0%	2.01	0.47–8.61	
Subarachnoid hemorrhage grade									
Good-grade (WFNS I–III)	90.7%	9.3%	1.00		51.6%	48.4%	1.00		0.09
Poor-grade (WFNS IV–V)	51.4%	48.6%	9.22	5.04–16.85	24.1%	75.9%	3.35	1.25–8.93	
Acute hydrocephalus									
No	88.8%	11.2%	1.00		51.2%	48.8%	1.00		0.07
Yes	62.9%	37.1%	4.68	2.61–8.41	36.5%	63.5%	1.82	0.79–4.19	
Thick and diffuse hemorrhage									
No	80.1%	19.9%	1.00		53.8%	46.2%	1.00		0.64
Yes	73.9%	26.1%	1.43	0.84–2.42	38.8%	61.2%	1.84	0.74–4.59	
Intracerebral hemorrhage									
No	84.9%	15.1%	1.00		45.2%	54.8%	1.00		0.05
Yes	60.4%	39.6%	3.69	2.13–6.38	38.7%	61.3%	1.30	0.54–3.14	
Intraventricular hemorrhage									
No	88.7%	11.3%	1.00		54.3%	45.7%	1.00		0.26
Yes	67.3%	32.7%	3.84	2.08–7.06	36.2%	63.8%	2.09	0.89–4.91	
Aneurysm treatment modality									
Endovascular	75.5%	24.5%	1.00		50.8%	49.2%	1.00		0.04
Surgical	78.6%	21.4%	0.84	0.50–1.42	29.4%	70.6%	2.48	1.01–6.09	
Aneurysm location									
Anterior circulation	79.0%	21.0%	1.00		44.0%	56.0%	1.00		0.42
Posterior circulation	64.3%	35.7%	2.09	1.04–4.18	38.9%	61.1%	1.24	0.43–3.53	

*WFNS*, World Federation of Neurosurgical Societies grading scale

Among the 398 patients who were alive at 1 week after admission and for whom outcome data was available, DCI was not associated with outcome in either group (OR 1.68, 95% CI 0.97–2.91 in the younger group, and OR 1.17, 95% CI 0.45–3.00 in the older group). Among the 388 patients who were alive at 2 weeks, chronic hydrocephalus was significantly associated with unfavorable outcome only in the younger patients (OR 4.73, 95% CI 2.60–8.59, and OR 2.50, 95% CI 0.99–6.33 in the older group), but the interaction with age group did not seem significant (*p* = 0.26).

### Risk factors for a 12-month mortality

Sex or CCI were not associated with 12-month mortality in either age group. Poor-grade SAH and IVH increased the risk of death in both groups. In contrast, acute hydrocephalus was associated with increased mortality only in the younger group. ICH or a thick and diffuse bleeding pattern was not associated with mortality in either group. In the younger patients, a posterior circulation aneurysm was associated with increased mortality compared to anterior circulation. Aneurysm treatment modality was not associated with mortality in either group.

In the 404 patients alive at 1 week after admission, DCI was not associated with mortality in either group. Likewise, in the 393 patients alive at 2 weeks, chronic hydrocephalus was not associated with mortality in either group. Results of the risk factor analyses for 12-month mortality are shown in Supplemental Table 1.

### Effect of age on functional outcome and mortality

In the multivariate logistic regression model without age group, higher CCI, poor-grade SAH, acute hydrocephalus, ICH, and posterior circulation aneurysm increased the odds of unfavorable outcome at 12 months (Table 3). This model had a Nagelkerke *R*^2^ value of 0.29. When we added age group to this model, belonging to the older age group was significantly associated with unfavorable outcome (OR 5.48, 95% CI 3.0–10.0, see Table 3), and the new model had a Nagelkerke *R*^2^ of 0.38. The difference between the Log-likehoods of the models was significant (Chi-square 33.35, *p* < 0.001), indicating that the new model was a better fit to the data.

**Table 3:** Results of the multivariate logistic regression analysis without and with age group. Odds ratios (OR) with 95% confidence interval (CI) for unfavorable outcome at 12 months after subarachnoid hemorrhage are shown

	Model without age group		Model with age group	
	OR	95% CI	OR	95% CI
CCI score 1^a^	2.60	1.42–4.75	1.88	0.98–3.58
CCI score at least 2^a^	4.13	1.67–10.23	3.21	1.23–8.37
Poor-grade SAH (WFNS IV–V)	3.24	1.88–5.60	4.46	2.46–8.09
Presence of acute hydrocephalus	2.05	1.18–3.56	2.09	1.17–3.74
Thick and diffuse hemorrhage	1.23	0.73–2.07	0.91	0.52–1.59
Intraventricular hemorrhage	1.50	0.84–2.67	1.53	0.84–2.81
Intracerebral hemorrhage	2.09	1.19–3.69	1.99	1.10–3.58
Posterior circulation aneurysm^b^	2.31	1.19–4.50	2.00	0.99–4.02
Older age group			5.48	3.02–9.96

^a^Compared to CCI score 0

^b^Compare to anterior circulation aneurysm

*WFNS*, World Federation of Neurosurgical Societies grading scale

Regarding 12-month mortality, the multivariate regression model without age group had a Nagelkerke R^2^ of 0.22. Higher CCI, poor-grade SAH, IVH, and a posterior circulation aneurysm were associated with increased risk of death. Adding age group to this model increased the Nagelkerke *R*^2^ to 0.32. The older patients had a significantly higher mortality risk when compared to the younger patients (OR 7.0, 95% CI 3.2–15.3). The difference between the Log-likehoods of the models was significant (Chi-square 26.06, *p* < 0.001). Detailed results of the multivariate logistic regression analyses regarding 12-month mortality are shown in Supplemental Table 2.

## Discussion

### Key findings

In this retrospective, single-center study, we were able to compare younger (under 70 years of age) and older (at least 70 years of age) patients with equal aSAH severity at admission treated at a tertiary center neuro-ICU. All patients received active aneurysm treatment and neurocritical care. In the younger group, 77% achieved a favorable outcome at 12 months, compared to 43% of the older group. Likewise, mortality was significantly lower in the younger group (7 vs. 27%). The prognosis of older good-grade patients matched that of younger poor-grade patients. However, even in the older group, 82% of survivors were able to live at home at 12 months. Notably, none of the younger patients and only one older patient with poor-grade SAH were in a vegetative state at 12 months.

In multivariate models, the addition of age increased the models’ explained variance significantly, and higher age was associated with both increased likelihood of unfavorable outcome as well as death at 12 months. This indicates that older age is an independent risk factor for unfavorable outcome and death at 12 months in hospitalized aSAH patients that receive active treatment.

Being in line with previous reports, the older patients were more often women [20]. Additionally, as indicated by their higher CCI scores, the older patients had more co-morbidities. However, the CCI scores of the older patients were low, suggesting that the patients admitted to our neuro-ICU represented a previously healthy group of elderly people with high pre-admission functional reserves. The bleeding pattern was more often thick and diffuse in the older patients, which was to be expected due to their larger cerebrospinal fluid spaces caused by brain atrophy. A thick and diffuse bleeding pattern is regarded a risk factor for DCI [9], but DCI requiring treatment was more seldom seen in the older patients. Some previous studies support this finding [28], but our finding could also reflect a more conservative treatment approach in the elderly. Limitations of care were more common in the elderly, and their neuro-ICU episodes were shorter. In line with previous studies [29], chronic hydrocephalus requiring a shunt procedure was more often seen in the elderly patients.

There were some differences in the prognostic factors for unfavorable outcome between age groups. Overall, poor-grade SAH (WFNS IV-V) was the strongest predictive factor for unfavorable outcome in both groups. The effect seemed stronger in the younger patients, probably reflecting the small number of younger good-grade patients with unfavorable outcome. Additionally, younger SAH patients seemed to be more intolerant towards increases in intracranial pressure, either due to acute hydrocephalus or ICH, as both showed a detrimental effect on outcome only in the younger group. Lastly, compared to endovascular treatment, surgical aneurysm closure was associated with unfavorable outcome and increased mortality in the elderly, but not in the younger SAH patients.

### Comparison with previous literature

Our findings are in line with recent prospective studies on elderly SAH patients. Catapano et al. reported functional independence in 42% of elderly patients [4]. In a study by Proust et al., favorable outcome was seen in 66% of elderly good-grade patients who had their aneurysm secured, and only in 17% of poor-grade patients [23]. Previous retrospective studies have reported a favorable outcome in 10–57% of elderly SAH patients [10, 16, 21]. Ryttlefors et al. compared the outcome between under and at least 65-year-old SAH patients requiring an EVD and saw favorable outcome in 43% and 24% of patients, respectively [26].

The prognostic factors for unfavorable outcome in our study were similar to those seen in large multicenter studies, with advanced age and WFNS grade affecting outcome the most [13, 24]. Few studies have compared risk factors of poor outcome between age groups. In contrast to our findings, Degos et al. found that acute hydrocephalus treated with an EVD was associated with unfavorable outcome only in older patients [6]. The contradictory findings may reflect different indications for EVD placement.

In our study, surgical aneurysm closure was associated with unfavorable outcome and mortality at 12 months only in the older age group. In contrast, most of previous studies have found no differences between surgical and endovascular treatment in the elderly [12, 22, 25].

### Strengths and limitations

We were able to compare actively treated elderly and younger aSAH patients matched on SAH severity. If we had included all aSAH patients admitted to our hospital, the results would have favored the younger patients, as aneurysm treatment was more often withheld in the elderly. The 3:1 matching of younger patients was done for all admitted patients, but the WFNS distribution between the age groups did not differ even when considering only patients, whose aneurysm was secured.

We had extensive data regarding the patients’ clinical and radiological characteristics on admission and also possible SAH complications (i.e., acute and chronic hydrocephalus, delayed cerebral ischemia). It is generally appreciated that recovery after a serious central nervous insult may take up to a year, and hence, we decided to assess our patients’ functional outcome (GOS) at 12 months. Even though this was done retrospectively, we believe that the GOS classification results are reliable, as we had access to vast communal medical records.

Our study has some limitations. Due to the retrospective study design, we could not rule out that the treating clinicians’ take on patients’ prognosis may have affected their treatment decisions, causing a self-fulfilling prophecy. The shorter stay duration in the neuro-ICU and greater number of limitation-of-care orders in the older patients could be an indication of this. Also, regarding risk factor assessment for unfavorable outcome and mortality after aSAH, this is solely a hospitalized cohort in a specialist ICU of a large tertiary university hospital, and thus, there is a risk of survival bias affecting the associations between the risk factors identified and outcome [19]. Our cohort of older patients represents previously rather healthy individuals, and our treatment results cannot be generalized to the overall population of over 70-year-olds with aSAH.

In the interaction analyses, even though we were able to include 412 patients in our study, the number of patients in some of the subgroups was small. Therefore, we considered a *p* value of <0.1 indicative of a possible interaction effect, but only if the odds ratios clearly differed between age groups.

## Conclusions

Overall, our study design allowed us to compare outcomes and prognostic factors between two age groups (i.e., under and at least 70 years of age) with aSAH who received active neurosurgical treatment and neurointensive care at a tertiary hospital. We found that older age is a significant, independent risk factor for poor outcome and death at 12 months. However, the most important prognostic factor for poor functional outcome and death at 12 months was WFNS score at admission, and this effect seemed even stronger in the younger patients. Additionally, ICH and acute hydrocephalus were associated with unfavorable outcome only in the younger patients.

## Supplementary Information

Supplemental Table 1 (PDF 123 kb)

Supplemental Table 2 (PDF 85 kb)
