# Supplementary material for: Neurointensive care results and risk factors for unfavorable outcome in aneurysmatic SAH: a comparison of two age groups
Source: Acta Neurochir (Wien). 2021 Jan 29;163(5):1469–78. doi: 10.1007/s00701-021-04731-4 (PMC8053651; doi:10.1007/s00701-021-04731-4)
Supplement: Supplementary file 1 — (PDF 123 kb) [file 701_2021_4731_MOESM1_ESM.pdf]

Supplemental Table 1

Results of the univariate logistic and interaction analysis for mortality at 12 months after subarachnoid hemorrhage. Proportion of alive and dead patients, as well as odds ratios (OR) with 95% confidence intervals (CI) for mortality are shown.

	Younger group (n = 317)				Older group (n = 95)				
	**Alive (%)**	**Dead (%)**	**OR**	**95 % CI**	**Alive (%)**	**Dead (%)**	**OR**	**95 % CI**	**Age group interaction (p)**
**Sex**									
Women	94.1 %	5.9 %	1.00		72.8 %	27.2 %	1.00		0.68
Men	91.5 %	8.5 %	1.48	(0.62-3.52)	71.4 %	28.6 %	1.07	(0.31-3.78)	
**CCI**									
0	92.9 %	7.1 %	1.00		76.4 %	23.6 %	1.00		0.70
1	93.6 %	6.4 %	0.89	(0.25-3.15)	65.5 %	34.5 %	1.70	(0.63-4.56)	
At least 2	94.1 %	5.9 %	0.82	(0.10-6.51)	70.0 %	30.0 %	1.39	(0.31-6.14)	
**SAH grade**									
Good grade (WFNS I-III)	97.1 %	2.9 %	1.00		84.6 %	15.4 %	1.00		0.89
Poor grade (WFNS IV-V)	85.5 %	14.5 %	5.70	(2.16-15.04)	46.7 %	53.3 %	6.29	(2.35-16.82)	
**Acute hydrocephalus**									
No	97.7 %	2.3 %	1.00		81.0 %	19.0 %	1.00		0.18
Yes	87.6 %	12.4 %	5.95	(1.97-18.02)	66.0 %	34.0 %	2.19	(0.84-5.69)	
**Thick and diffuse hemorrhage**									
No	92.4 %	7.6 %	1.00		84.6 %	15.4 %	1.00		0.13
Yes	93.7 %	6.3 %	0.82	(0.34-1.95)	68.1 %	31.9 %	2.57	(0.79-8.37)	
**Intracerebral hemorrhage**									
No	94.4 %	5.6 %	1.00		73.4 %	26.6 %	1.00		0.49
Yes	90.4 %	9.6 %	1.78	(0.74-4.27)	71.0 %	29.0 %	1.13	(0.44-2.94)	
**Intraventricular hemorrhage**									
No	97.9 %	2.1 %	1.00		91.7 %	8.3 %	1.00		0.82
Yes	89.1 %	10.9 %	5.72	(1.66-19.75)	61.0 %	39.0 %	7.03	(1.93-25.60)	
**Aneurysm treatment modality**									
Endovascular	91.3 %	8.7 %	1.00		78.0 %	22.0 %	1.00		0.05
Surgigal	94.9 %	5.1 %	0.57	(0.23-1.39)	63.9 %	36.1 %	2.00	(0.80-5.01)	
**Aneurysm location**									
Anterior circulation	90.3 %	9.7 %	1.00		74.0 %	26.0 %	1.00		0.13
Posterior circulation	76.7 %	23.3 %	4.39	(1.72-11.22)	66.7 %	33.3 %	1.43	(0.47-4.30)	
**Delayed cerebral ischaemia**[a]									
No	92.9 %	7.1 %	1.00		72.1 %	27.9 %	1.00		0.98
Yes	96.2 %	3.8 %	0.53	(0.19-1.46)	83.3 %	16.7 %	0.516	(0.16-1.71)	
**Chronic hydrocephalus**[b]									
No	95.7 %	4.3 %	1.00		76.4 %	23.6 %	1.00		0.44
Yes	95.8 %	4.2 %	0.98	(0.26-3.66)	87.1 %	12.9 %	0.479	(0.14-1.62)	

[a] Only patients alive at one week. 312 patients in the younger group and 92 patients in the older group

[b] Only patients alive at one week. 307 patients in the younger group and 86 patients in the older group
