# Supplementary material for: Neurointensive care results and risk factors for unfavorable outcome in aneurysmatic SAH: a comparison of two age groups
Source: Acta Neurochir (Wien). 2021 Jan 29;163(5):1469–78. doi: 10.1007/s00701-021-04731-4 (PMC8053651; doi:10.1007/s00701-021-04731-4)
Supplement: Supplementary file 2 — (PDF 85 kb) [file 701_2021_4731_MOESM2_ESM.pdf]

Supplemental Table 2

Results of the multinomial logistic regression analysis without and with age group. Odds ratios (OR) with 95 % confidence interval (CI) for mortality at 12 months after subarachnoid hemorrhage are shown.

	**Multinomial model without age group**		**Multinomial model with age group**	
	**OR**	**95 %**	**OR**	**95 % CI**
CCI score 1[a]	2.19	(1.01-4.74)	1.45	(0.63-3.33)
CCI score at least 2[a]	2.82	(0.83-9.56)	1.85	(0.49-6.96)
Poor grade SAH (WFNS IV-V)	3.56	(1.64-7.73)	5.02	(2.15-11.75)
Presence of acute hydrocephalus	1.62	(0.73-3.58)	1.77	(0.77-4.09)
Thick and diffuse hemorrhage	0.91	(0.44-1.86)	0.56	(0.25-1.23)
Presence of intraventricular hemorrhage	3.25	(1.21-8.74)	3.42	(1.26-9.30)
Presence of intracerebral hemorrhage	0.95	(0.43-2.11)	1.04	(0.46-2.37)
Posterior circulation aneurysm[b]	2.42	(1.06-5.50)	2.35	(1.00-5.52)
Older age group			7.04	(3.25-15.27)

[a] Compared to CCI score 0

[b] Compared to anterior circulation aneurysm
